# The effect of fixed appliances on oral malodor from beginning of treatment till 1 year

**DOI:** 10.1186/s12903-016-0174-3

**Published:** 2016-02-04

**Authors:** Oral Sökücü, Aysun Akpınar, Hakan Özdemir, Muhammet Birlik, Metin Çalışır

**Affiliations:** Department of Orthodontics, Faculty of Dentistry, University of Gaziantep, Gaziantep, Turkey; Department of Periodontology, Faculty of Dentistry, University of Cumhuriyet, Sivas, Turkey; Department of Periodontology, Faculty of Dentistry, University of Osmangazi, Eskişehir, Turkey; Department of Orthodontics, Faculty of Dentistry, University of Bezmialem, Istanbul, Turkey; Department of Periodontology, Faculty of Dentistry, University of Adıyaman, Adıyaman, Turkey; Department of Orthodontics, Faculty of Dentistry, Bezmialem Vakıf University, 34093 Istanbul, Turkey

**Keywords:** Fixed orthodontic treatment, Halitosis, Oral malodor, Halimeter, Volatile sulfide compounds

## Abstract

**Background:**

Orthodontic appliances can enhance plaque accumulation, and this can cause gingival inflammation. Halitosis of oral origin is associated with microbial metabolism on the tongue and in the saliva, dental plaque, and the amount of volatile sulfide-containing compounds. This study used a Halimeter to investigate fixed orthodontic therapy-associated increases in the oral malodor over a year.

**Methods:**

Thirteen orthodontic patients with Angle Class I malocclusions receiving fixed orthodontic therapy formed the study group, and 12 dental students without any dental treatment formed the control group. The Halimeter was used to examine oral malodor by detecting volatile sulfur compounds (VSC). Plaque index (PI), gingival index (GI), and probing pocket depth (PPD) were also measured in both groups. The subjects in the study group had one visit before the orthodontic treatment started and seven visits during orthodontic therapy (1, 3, 5, 7, 9, 11, and 13 months after bonding), while the subjects in the control group had three visits, once per subsequent month.

**Results:**

Oral malodor was significantly increased in the fixed orthodontic treatment group during treatment (*p* < .05). Increases were also observed in the PI, GI, and PPD measures (*p* < .05). The results of the control group were stable (*p* > .05).

**Conclusion:**

Oral malodor increased during fixed orthodontic treatments and reached a critical level 7 months later.

## Background

Dentition irregularities and disrupted relationships of the dental arches are defined as malocclusions. Disorders in the positions of the teeth have been suggested to increase dental plaque accumulation, thus exposing the patient to periodontal disease and the formation of caries. It has been widely suggested that straighter teeth are easier to clean and may promote a healthier periodontium [1].

In treating malocclusions, fixed orthodontic therapy is the first choice. Although one of the goals of orthodontic treatment in subjects with malocclusions is to improve their periodontal health, the therapy itself may result in an increased incidence of gingival bleeding, recession, and plaque retention [2]. Since the surface roughness and high surface-free energies are positively correlated with plaque accumulation and maturation [3], the presence of orthodontic appliances can enhance the plaque accumulation and, eventually, cause gingival inflammation in subjects with malocclusion [4]. It is well documented that orthodontic treatment with fixed appliances is accompanied by an increased risk of gingivitis due to the accumulation of bacterial plaque around the attachments [3, 4].

Halitosis is a lyrical term derived from the Latin *halitus* (breath), and the Greek suffix *osis* (condition, action of a pathological process) [5]. Halitosis of oral origin is associated with microbial metabolism on the tongue dorsum, in the saliva, and dental plaque [6]. The intensity of clinical bad breath is significantly associated with the amount of intraoral volatile sulfide-containing compounds. These compounds are produced by gram-negative oral bacteria that metabolize amino acids in the diet and produce gases, such as hydrogen sulfide (H_2_S) [7]. There are three generally accepted methods for the assessment of oral malodor: organoleptic measurement, gas chromatography (GC), and portable sulfide monitoring [8]. A portable Halimeter (Halimeter, Interscan Corp., Chatsworth, CA) is used to measure volatile sulfide compounds (VSC).

The long-term influence of orthodontic appliances has been shown to change dental plaque accumulation. While the short-term effects of oral malodor in fixed orthodontic therapy have been evaluated, the long-term effects have not yet been documented [9–12].

Our hypothesis was that orthodontic treatment may affect oral malodor, especially in subjects with malocclusion. The aim of the present longitudinal study was to examine the impact of fixed orthodontic therapy on periodontal status and oral malodor in a population with malocclusions. For the purposes of this study, the null hypothesis assumed that orthodontic treatment does not adversely affect periodontal status and does not increase oral malodor.

## Methods

### Study population

Ethical approval (B.30.2.CUM.0.04.00.00/99) was obtained for this study from the Human Research Ethics Committee at the Cumhuriyet University School of Medicine in Sivas. Informed consent was obtained from all of the subjects, and the study protocol was approved by the Medical Ethics Committee of Cumhuriyet University. The volunteers and controls for this study participated between April 2008 and December 2009. The inclusion criteria for this study were: all subjects have brushing habits of at least two times per day, are in good general health, have no recent medications, and no previous orthodontic treatment. Exclusion criteria were: unsatisfactory oral hygiene, antibiotic use during or up to 4 months prior to the study, smoking, having an orthognathic surgery requirement, a self-declared history of systemic diseases, medicine use, and an unwillingness to participate in the study. The participants were asked to abstain from eating and drinking for at least 8 h prior to the visits, but were allowed to drink water until 3 h before the examinations. Subjects were also asked to refrain from their normal oral hygiene practices and from the use of oral rinses and breath fresheners, and to abstain from smoking 12 h prior to the assessments.

Patients were also requested to avoid spicy foods, onions, and garlic 48 h prior to the appointment, and they were not allowed to use scented cosmetics or aftershave lotions on the day of the appointment. Furthermore, all subjects had to have at least 20 teeth.

Twenty subjects with Angle Class I malocclusions, who were willing to participate in the present study, were recruited for the case group. Class I patients were selected because this group consists of orthodontic patients that did not have severe craniofacial anomalies and, therefore, were able to finish the study in 1 year. Seven subjects did not continue to participate during the first three months of the follow-up period due to personal reasons, and they were excluded from the study. Thirteen patients (eight females and five males) formed the final case group. Twelve dental students (seven females and five males) who did not receive orthodontic treatment formed the control group. Before the study began, all of the subjects received oral hygiene instructions from a periodontist. After this instruction, the orthodontist informed the subjects of the details of this study.

The subjects in the case group had one visit before the orthodontic treatment began and seven visits during orthodontic therapy (1, 3, 5, 7, 9, 11, and 13 months after bonding). The subjects in the control group had three visits, once per subsequent month. The patients were instructed to continue ideal oral hygiene; if not, they were informed again to maintain it. Unfortunately, due to the failure of cooperation, these patients were excluded from the study.

### Orthodontic treatment

Orthodontic therapy consisted of molar bands (convertible triple tube, 0.018 × 0.025, American Orthodontics Inc., Sheboygan, Wisconsin, USA) with edgewise triple buccal tubes with vertical hooks. Also, in the second and first premolars, canines, and lateral and central incisors, direct-bonded brackets (Roth Mini Master Brackets, American Orthodontics) were used. The maxillary teeth were leveled with continuous archwires, starting with 0.012-inch nickel-titanium, and working up to 0.016 × 0.022-inch stainless steel (Ni-Ti Memory Wire, force 1, form 3, and stainless steel wire archwires, American Orthodontics). The therapy was applied by the same orthodontist (M.B.) to all patients.

### Periodontal examination

Each subject was examined by a single periodontist (A.A.) who carried out the periodontal examinations throughout the study. Clinical periodontal parameters were collected by determining the Plaque Index (PI) [13] (Table 1), the Gingival Index (GI) [14] (Table 2), and the Probing Depth (PPD) (Table 3) scores.

**Table 1:** Mean values and standard deviations of plaque index (PI) measurements, before the orthodontic treatment had started (0), and at every second month (1, 3, 5, 7, 9, 11, 13) during the treatment. Since the index levels of the control group were steady during the three visits, one mean value is given

Plaque index	Initial	1st month	3rd month	5th month	7th month	9th month	11th month	13th month	*Control group*
Mean	0.626^a^	0.644^a^	0.715^a^	0.782^a^	0.786^a^	0.624^a^	0.724^a^	0.795^a^	*0.280*
Standard deviation	0.114	0.140	0.138	0.108	0.124	0.118	0.120	0.133	*0.083*

^a^Significant

**Table 2:** Mean values and standard deviations of gingival index (GI) measurements, before the orthodontic treatment had started (0), and at every second month (1, 3, 5, 7, 9, 11, 13) during the treatment

Gingival index	Initial	1st month	3rd month	5th month	7th month	9th month	11th month	13th month	*Control group*
Mean	0.815^a^	0.862^a^	1.014^a^	0.962^a^	1.166^a^	0.841^a^	0.962^a^	0.975^a^	*0.33*
Standard deviation	0.184	0.189	0.260	0.172	0.157	0.126	0.106	0.160	*0.123*

^a^Significant

**Table 3:** Mean values and standard deviations of probing pocket depth (PPD) measurements, before the orthodontic treatment had started (0), and at every second month (1, 3, 5, 7, 9, 11, 13) during the treatment

Pocket depth	Initial	1st month	3rd month	5th month	7th month	9th month	11th month	13th month	*Control group*
Mean	1.976^a^	2.050^a^	1.914^a^	2.039^a^	2.378^a^	2.273^a^	2.219^a^	2.294^a^	*1.77*
Standard deviation	0.294	0.280	0.251	0.320	0.254	0.308	0.157	0.076	*0.131*

^a^Significant

During the study, the participants continued their usual oral hygiene practices, and all patients used the same assigned brushing method (the Bass technique) and toothbrushes (Oral-B Pro-Expert 3DClean). The oral hygiene instructions were repeated at 1, 3, 5, 7, 9, 11, and 13 months. For each examination, the subjects were instructed to brush their teeth after dinner and to refrain from eating and drinking until coming to the dentistry faculty the next morning.

### Oral malodor

In this study, a portable Halimeter (Halimeter, Interscan Corp., Chatsworth, CA, USA) was used to detect the oral malodor. Each patient kept their mouth closed for 60 s prior to the measurement. A plastic straw was inserted into patient’s mouth placed above the dorsal portion of the tongue, and not touching the oral mucosa or the tongue. Breathing was not allowed during the measurement, and the participant was asked not to exhale or inhale during the Halimeter reading. Measurements were duplicated and the mean values were calculated (Table 4).

**Table 4:** Mean values and standard deviations of oral malodor measurement, before the orthodontic treatment had started (0), and at every second month (1, 3, 5, 7, 9, 11, 13) during the treatment. Since the index levels of control group were steady during the three visits, one mean value is given

Oral malodor	Initial	1st month	3rd month	5th month	7th month	9th month	11th month	13th month	*Control group*
Mean	115.92^a^	139.56^a^	145.48^a^	147.54^a^	152.46^a^	135.64^a^	137.67^a^	139.70^a^	*70.50*
Standard deviation	8.716	8.741	7.650	6.90	6.130	5.20	6.760	7.824	*8.220*

^a^Significant

Oral malodor values were divided into four categories and classified as normal (for values ranging from 0 to 100 parts per billion [ppb]), weak (101 to 150 ppb), strong (151 to 300 ppb), or very strong (≥301 ppb) [15].

### Statistical analysis

The SPSS statistical program (Version 14.0; SPSS Inc., Chicago, IL, USA) was used to analyze the data, and the paired-samples t-test was used for the comparisons of the clinical parameters between each visit. Furthermore, the independent-samples t-test was used for the comparison between the groups. Pearson rank correlation analyses were used to analyze the correlations between the clinical parameters, and *p* values < .05 were considered statistically significant.

## Results

The mean values and standard deviations of the clinical measurements of oral malodor, PI, GI, and PPD, are given in Tables 1, 2, 3, and 4. The PI of the study group was stable from the beginning until the final measurement. However, the PI was significantly higher in the study group than in the control group (*p* < .05). GI scores showed the best scores on the initial records. In the months subsequent to the fixed orthodontic treatment, the GI scores increase in the study group and were significantly higher than in the control group (*p* < .05). PPD was the lowest during the initial records. The PPDs increased after the fixed orthodontic treatment procedures and the scores in the study group were significantly higher than in the control group (*p* < .05). All of the study groups’ measured parameters were significantly higher than in the control group (*p* < .05). Thus, the null hypothesis was rejected.

Oral malodor began to increase from the base measurement until the 7th measurement (152 ppb), which can be seen in Table 4. All of the malodor scores were significantly higher in the study group than in the control group (*p* < .05).

## Discussion

In the present study, changes in oral malodor and periodontal status were followed over 1 year in a study population that received fixed orthodontic therapy. Subjects without malocclusion and orthodontic therapy served as the control group.

The most common cause of oral malodor is elevated levels of VSCs, primarily H_2_S and methyl mercaptan (CH_3_SH), in the breath. In this study, a portable sulfide monitor was used to measure the VSCs. The advantages of this monitor included the ease of use by non-skilled individuals, non-invasiveness, low possibility for cross-infection, portability, relatively inexpensive, and it offered a rapid turnaround time of 1 to 2 min between measurements [16].

VSC levels in the mouth correlate with the depth of the periodontal pockets, and the amount of VSCs in the breath increase with the number, depth, and bleeding tendency of these periodontal pockets [7].

According to our clinical observations, fixed orthodontic therapy causes an increase in dental plaque accumulation, gingival inflammation, and PPDs. As no attachment loss was observed, the increased PPD scores refer to pseudo-pocket formations due to gingival overgrowth.

After the application of the brackets, the oral malodor scores began to increase over the next seven months. It was interesting that oral malodor scores at the beginning of the treatment without brackets in the study group were also higher than in the control group. Buckley [17] and Bollen [18] demonstrated that there is a significant relationship between irregular dentition and periodontal disease. Subjects with malocclusions have a higher tendency for increased dental plaque accumulation, gingival inflammation, and PPDs than those with normal occlusions. In the present study, the increase in oral malodor may correlate with the increased pocket depths, which may stem from the inflammation in the gingival and other periodontal tissues, poor salivary flow, excessive dental plaque accumulation, or unclean dental appliances.

Neiders and Ramos [19] stated that the oral microbial metabolism of the plaque, on the tongue dorsum, or in the periodontal pockets could help to cause bad breath. Eight to 14 h of maturation are required before plaque deposits produce VSCs. Protected plaque in the interproximal sites produce substantial odors and is associated with the overall VSC levels. Babacan et al. [9] observed orthodontic patients for 4 weeks and found a significant increase in oral malodor with the same measurement method. They concluded that the presence of PI and GI could cause conditions suitable to oral malodor. Alternatively, Tonzetich [20] found that fixed orthodontic therapy did not cause oral malodor, but there was a correlation between plaque and oral malodor.

Fixed appliances may restrict the ability of the tongue to remove food particles from the mouth. The presence of carbohydrates gives rise to a prolonged acid challenge to the tooth, and encourages the growth of aciduric bacteria, such as mutans streptococci and lactobacilli. A fivefold increase in lactobacillus counts has been observed in patients that underwent active orthodontic treatment; therefore, many growth areas were detected on the gingival margins and on the edges of the orthodontic bands [21].

One could speculate on the small sample size being a limitation in this study. However, the present study contributes to the literature with its long-term design since there are few studies on the effects of orthodontic therapy on periodontal status where the follow-up has continued for up to 1 year or more [21, 22].

The initial oral malodor score was detected as 115 ppb, and 1 year after treatment this increased to 139 ppb. The oral malodor scores reached the critical level 7 months later. Therefore, the motivation of the orthodontic patient to maintain periodontal health is important. The increase in oral malodor stopped during the ninth month of orthodontic treatment, and thereafter, the oral malodor scores were stable in the orthodontic patients. These results suggest that orthodontic treatment causes an increase in oral malodor over a limited period of time, which may depend on the total microbial load of the oral flora [7]. The retentive areas consisting of brackets and archwires may stimulate an increase in the VSC compounds causing oral malodor. The other parameters of the periodontium include PI, GI, and PPD scores that increase at the same time. These parameters may also affect the oral malodor scores.

One significant result of this study is that oral malodor may be added as an indicator to checking the oral periodontium of patients.

The control group of patients also showed stable results on all measurement parameters.

## Conclusion

- Orthodontic treatment affects oral malodor with regard to the PI, GI, and PPD.
- The critical limit of oral malodor was reached at the end of 7 months.
- Oral malodor can be used as an indicator to evaluate the oral health of patients.
- Clinically, after 7 months of fixed orthodontic treatment, the clinician will follow not only the GI and PI, but also the oral malodor to establish ideal oral health in patients.

## Abbreviations

GC: gas chromatography
GI: gingival index
PI: plaque index
ppb: parts per billion
PPD: probing pocket depth
VSC: volatile sulfur compounds
